# Molecular cross talk among the components of the regulatory machinery of mitochondrial structure and quality control

**DOI:** 10.1038/s12276-020-0434-9

**Published:** 2020-05-12

**Authors:** Hyo Min Cho, Woong Sun

**Affiliations:** Department of Anatomy, Korea University College of Medicine, Brain Korea 21 plus, Seoul, 02841 Republic of Korea

**Keywords:** Organelles, Autophagy

## Abstract

Mitochondrial dysfunction critically impairs cellular health and often causes or affects the progression of several diseases, including neurodegenerative diseases and cancer. Thus, cells must have several ways to monitor the condition of mitochondrial quality and maintain mitochondrial health. Accumulating evidence suggests that the molecular machinery responding to spontaneous changes in mitochondrial morphology is associated with the routine mitochondrial quality control system. In this short review, we discuss recent progress made in linking mitochondrial structural dynamics and the quality control system.

## Mitochondria: shape shifts provide clues to cellular health

The health of mitochondria is important for cellular health, and is maintained by the same mechanisms that control their shape. Mitochondria continuously divide, fuse, elongate, and shrink, forming ever-changing networks inside cells. Damaged mitochondria produce toxic byproducts and have been implicated in neurodegenerative diseases and cancer. Although changes in mitochondrial structure are known to be related to cellular health, the detailed mechanisms are not well understood. In a review, Woong Sun and Hyo Min Cho at the Korea University College of Medicine, Seoul, detail how mitochondrial fusion, division, and recycling are controlled, what signals are used to dispose of damaged mitochondria, and how the shape-control mechanisms also regulate mitochondrial quality. This review will help us to more clearly understand the structure-function relationship of mitochondria.

## Introduction

Mitochondria are organelles with multiple functions for cell life, including the production of ATP for the generation of cellular energy through the electron transport chain (ETC), buffering cytosolic ions such as iron, calcium, and zinc, metabolizing specific lipids and amino acids, providing compartments for isolating molecules such as apoptosis-inducing factor, and generating heat^1^. To accomplish these diverse and complex functions, mitochondria are enclosed by a unique double membrane with specialized and functionally compartmentalized structures. These unique structures have emerged during the adaptive evolution of endosymbiotic alphaproteobacteria^2,3^. According to this endosymbiosis theory, the outer mitochondrial membrane (OMM) is derived from endocytic vesicles in the cytosol, and the inner mitochondrial membrane (IMM) originates from bacteria. In this respect, the IMM underwent greater morphological changes with the emergence of cristae structures, which increased the membrane surface area to support ETC reactions for ATP synthesis^4^. To produce and maintain cristae structures, many host-derived proteins are imported into the mitochondria and form specialized spots for junction formation^4^.

Another important feature of mitochondrial morphology is its dynamic nature. Both OMM and IMM, separately or simultaneously, can change their morphology via fission, fusion, and other forms of morphological alteration^5^. Mitochondrial morphology is primarily determined by the balance between mitochondrial fusion and fission^6^. In a steady state, mitochondria continuously repeat fusion–fission cycles to maintain their morphology under dynamic equilibrium. When the cell environment changes or when appropriate stimuli are perceived, the mitochondrial morphology is shifted to a new equilibrium, acquiring either a hyperfragmented or a hyperconnected status. The physiological stimuli affecting mitochondrial morphology are highly diverse and include the metabolic status of the cell, growth signals, cell cycle- and circadian rhythm-related signals, apoptosis, and various forms deleterious stress^6^. These morphological dynamics require the consumption of cellular energy, and the perturbation of this process can cause diverse diseases, indicating that the control of mitochondrial morphology is pivotal for the maintenance of cellular health. Although the correlation between mitochondrial structural changes and cellular health has been extensively studied, the precise mechanisms underlying this relationship remain incompletely understood. However, some experimental data suggest that mitochondrial morphological alterations are related to the mitochondrial quality control system. In this short review, we discuss the fundamental mechanisms of (1) how mitochondrial structures are molecularly modulated, (2) how mitochondrial quality is monitored and controlled, and (3) how these two mechanisms are coordinated and interact at the molecular level. Finally, we discuss issues that should be considered when undertaking future research.

### Molecular regulation of mitochondrial structures

Mitochondrial morphology is determined by the balance between mitochondrial fusion and fission. Dynamin-related proteins (DRPs) are core proteins regulating morphological changes in mitochondria^7^. As their name indicates, these proteins are structurally similar to dynamin, which is critical for cellular vesicle formation. This fact indicates that the machinery controlling mitochondrial dynamics originated from that crucial for vesicle processing. Mitochondrial fusion and fission are controlled by different members of the DRP family, mainly mitofusin 1/2 (Mfn1/2) and optic atrophy 1 (Opa1), for membrane fusion, and dynamin-related protein 1 (Drp1) for fission. Mfn1/2 is located on the OMM, where it interacts with adjacent mitochondria through homo- or heterodimerization, and promotes mitochondrial fusion by GTP hydrolysis. OMM fusion is a multistep process, proceeding through at least three different steps^8^: (1) the OMMs from two opposing mitochondria are tethered through the interaction of Mfns in trans, (2) GTP binding induces conformational changes in the Mfns, which progressively increases the contact surface area and reduces the gap between the two membranes^8^, and (3) a GTPase-dependent power stroke or oligomerization completes OMM fusion^9,10^. On the other hand, IMM fusion is mediated by OPA1. Because OPA1 interacts with Mfn1/2 to form an intermembrane protein complex, which allows the coordinated fusion of IMM and OMM together, IMM fusion occurs after OMM fusion as a downstream event. However, OPA1 is sufficient to drive the fusion of both membranes in vitro^11^, and the requirement of Mfns for OPA1 activation is not yet clearly understood. IMM fusion also requires the IMM-specific lipid cardiolipin (CL). CL, which constitutes 15–20% of the IMM, makes conical structures by dimerization and strongly interacts with proteins. These characteristics of CL allow the organization of the cristae structures and oxidative phosphorylation (OXPHOS) complexes. The interaction between OPA1 and CL promotes the tethering of two IMMs via GTP hydrolysis. Considering that the CL is important for the mitochondrial contact site and cristae organizing system, a growing body of evidence also suggests that components of the mitochondrial fusion machinery, especially OPA1, is involved in cristae remodeling. OPA1 cleavage adds more complexity to the regulation of IMM fusion. OPA1 has proteolytic cleavage sites recognized by mitochondrial metalloproteases, such as overlapping with the M-AAA protease 1 homolog and YME1-like ATPase, which generate soluble OPA1 fragments (S-OPA1). This cleavage is regulated by cellular stress or OXPHOS status, suggesting that it has regulatory functions in the stimulus-dependent pathway of mitochondrial fusion. However, the role of S-OPA1 is not yet fully understood, and its exact function in regulating mitochondrial morphology is somewhat controversial^11–17^.

Drp1 is a major player during mitochondrial fission^18–20^. For fission to occur, cytosolically localized Drp1 monomers or dimers translocate to the outer surface of the mitochondria via interactions with Drp1 receptors located in the OMM. The mitochondrial recruitment of Drp1 is tightly controlled by Drp1 posttranslational modifications, such as phosphorylation, SUMOylation, S-nitrosylation, and glycosylation^17^. A ring-like structure is then formed via Drp1 self-oligomerization, which can sever the mitochondrion via scissor-like structural alterations driven by GTP hydrolysis. Live imaging using GFP-Drp1-expressing cells showed that mitochondria are not cut immediately upon the translocation of GFP-Drp1^21^, suggesting that mitochondrial fission proceeds through multiple steps. In fact, a growing body of evidence supports the hypothesis that mitochondria are severed by multiple OMM and IMM constrictions because their diameter is sufficiently large that cleavage cannot be completed by only one protein complex. Furthermore, although purified Drp1 has the ability to constrict lipidic structures, it fails to sever them in vitro^22–24^, indicating that additional factors are required for lipid membrane severing. In fact, the diameter of the Drp1 ring structure (<110 nm) is much smaller than the average diameter of a mitochondrion (~1 µm), and mitochondrial preconstriction is necessary for Drp1 ring formation. Interestingly, most mitochondrial fission (88%) occurs at contact sites between mitochondria and the endoplasmic reticulum (ER), where the mitochondrial diameter is sufficiently small (138–146 nm) to allow cutting by the Drp1 ring^25^. Mitochondrial depression at ER contact sites is mediated by actin polymerization. For example, destabilization of actin filaments prevents OXPHOS inhibition-induced mitochondrial fragmentation, which is dependent on Drp1 activity^26^. It has been proposed that inverted formin 2 (INF2) is critical for mitochondrial preconstriction at the ER contact sites since it is localized at the ER and can induce mitochondrial membrane constriction by interacting with the mitochondrial actin-nucleating protein spire1C^25,27,28^. However, INF2-dependent actin polymerization appears to be insufficient to promote mitochondrial constriction, and other actin-remodeling molecules, such as Arp2/3, cofilin, and cortactin, also play important roles in Drp1-dependent mitochondrial fragmentation^29–32^. Actin filaments can generate a pulling force^33^ through coordinated interactions with the motor protein nonmuscle myosin II (NMII)^34–36^. For the execution of the last step of membrane cutting, dynamin2 appears to play a role^37^. However, deletion of dynamin family proteins (dynamin1, 2, or 3) cannot block mitochondrial fission, and controversy about the contribution of dynamins to mitochondrial fission remains^17^.

**Fig. 1:**
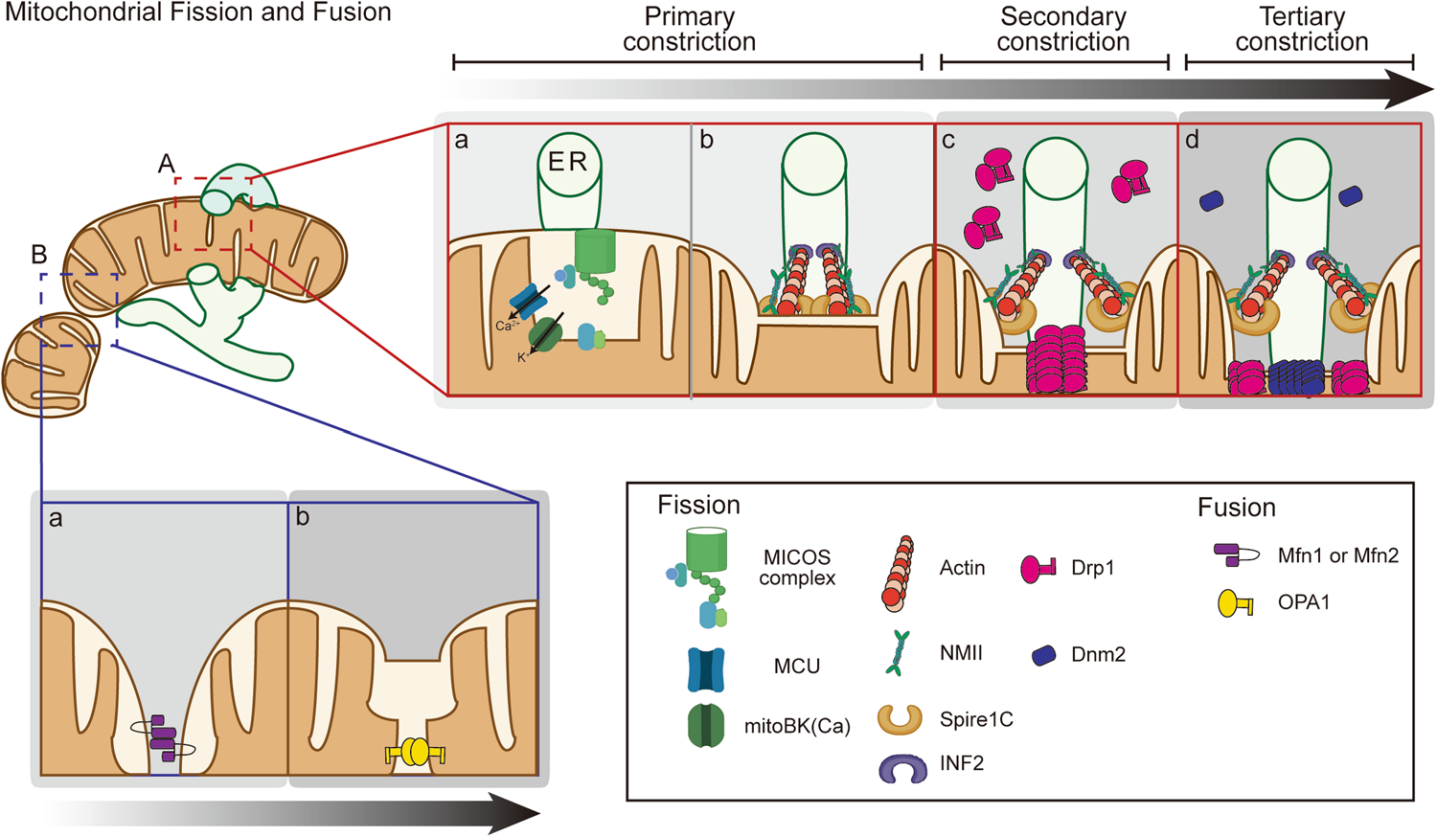
Mechanism of mitochondrial fission and fusion. **a** A mitochondrion is divided via multiple constrictions of the OMM and IMM. A The majority of mitochondrial fission takes place at contact sites between the mitochondria and the ER, which act as platforms for the accumulation of various proteins, such as actin, ion channels, and motor proteins, to initiate the constriction of the OMM and/or IMM. INF2 and spire1C induce actin polymerization on the ER side and mitochondrion side, respectively, allowing for the generation of an MNII-mediated pulling force. B IMM constriction proceeds independent of actin polymerization and Drp1 activation. It is required for the MCU-dependent Ca^2+^ influx that destabilizes the mitochondrial inner structure. Ca^2+^ influx activates mitoBK(Ca), which induces mitochondrial bulging. C Cytosolic Drp1 forms a ring structure at the constricted site, which further constricts the mitochondrion by GTP hydrolysis. D Dnm2 finally constricts the mitochondrion to a diameter of <10 nm, which is sufficient for lipid autocleavage. **b** Mitochondrial fusion is mediated by dynamin-related proteins, such as Mfn1/2 and OPA1. When two mitochondria approach each other, Mfn docking is initiated. The interaction between Mfns in their non-GTP binding state reduces the gap between the mitochondria. Then, GTP hydrolysis drives membrane fusion. Because OPA1 interacts with Mfns, it may promote IMM fusion following OMM fusion. Oligomerization and GTP hydrolysis promote conformational changes that bring the two membranes into close proximity, facilitating IMM fusion.

It is difficult to imagine that OMM constriction can sever the entire mitochondrion, including the IMM. A ring structure made by filamenting temperature sensitive mutant Z (FtsZ) is used for prokaryotic cell division^38^. In this respect, it is important to note that orthologs of FtsZ in primitive red algae contribute to the constriction of the IMM before the Drp1-dependent constriction of the OMM^39^. Although mammalian orthologs of FtsZ have not been found, IMM constriction has been reported in various mammalian models^19,20,40–42^. We have previously found that the constriction of the mitochondrial inner compartment (CoMIC) is independent of Drp1-dependent OMM fission. CoMIC occurs at the contact sites between the mitochondria and the ER and is dependent on increased levels of mitochondrial calcium. Currently, the molecular mechanisms facilitating CoMIC are unclear, but several candidate molecules affecting IMM morphology have been reported^43–46^. Interestingly, INF2-induced actin polymerization increases the levels of mitochondrial calcium in a mitochondrial calcium uniporter (MCU)-dependent manner^47,48^. Thus, calcium release initiated in the ER can promote both CoMIC and Drp1 induction^49,50^. In summary, mitochondrial fission does not occur in one step, and many processes (actin-dependent OMM constriction, Drp1-dependent OMM severing, Drp1-independent CoMIC, and Dyn2-dependent fission) have been identified thus far (Fig. 1). The precise relationship between these events and their relative contributions to mitochondrial fission should be investigated in the future.

### Multiple systems of mitochondrial quality control

**Fig. 2:**
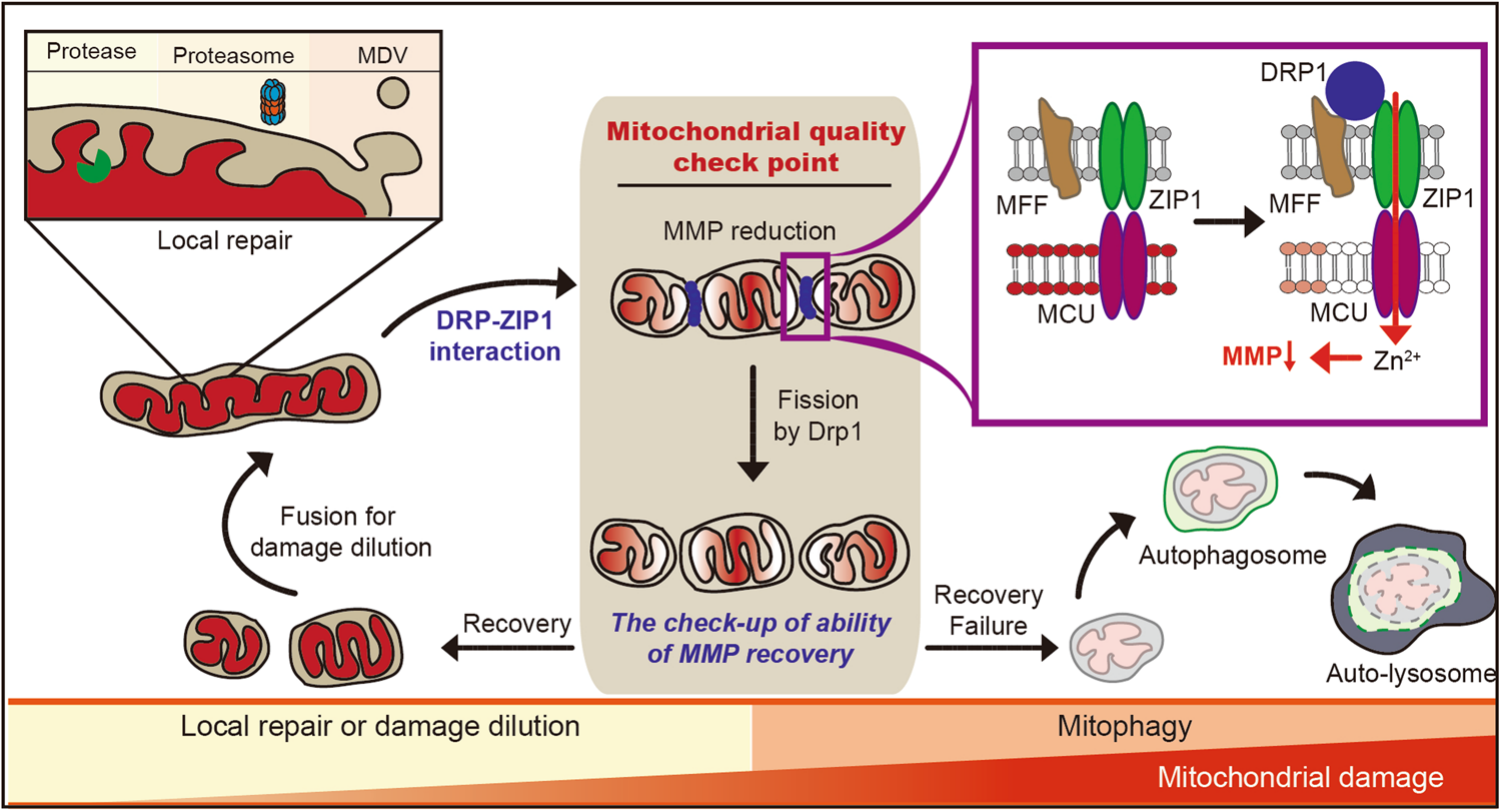
Mitochondrial quality control systems in cells. Mitochondrial proteases or proteasomes can remove damaged proteins from the mitochondria. In some cases, a small portion of a mitochondrion containing damaged proteins is segregated as a vesicle (MDV) and transferred to the lysosome. Massive mitochondrial damage that induces a reduction in mitochondrial membrane potential (MMP) triggers mitophagy. Under physiological conditions, the mitochondrial fission protein DRP1 focally reduces the MMP at the mitochondrial fission site via interactions with the mitochondrial zinc transporter ZIP1. This DRP1-induced MMP reduction serves to discriminate and selectively remove only damaged mitochondrial fragments through the localized induction of the mitophagy cascade.

The accumulation of dysfunctional mitochondria is deleterious for cellular health; therefore, it has been considered a causal factor for various diseases, such as neurodegenerative and cardiac diseases^51,52^. There are at least three different proteolytic quality control systems to maintain mitochondrial health: the ubiquitin-proteasome, mitochondria-derived vesicles/lysosomes, and autophagy (Fig. 2). Mitochondrial DNA exclusively encodes only 13 proteins associated with the ETC complex. Thus, most mitochondrial proteins are encoded by the nuclear genome and need to be imported after being produced in the cytosol. Transporting proteins from the cytosol into the mitochondria is a complex process requiring the penetration of both the OMM and IMM, and transport failure often triggers mitochondrial stress responses, involving the arrest of mitochondrial protein transport and the activation of the mitochondrial proteasome pathway to correct for the misfolded/aggregated proteins by several mechanisms, such as SUMOylation and ubiquitination-dependent proteasome activation^53–55^. Interestingly, mitochondria can serve as compartments for clearing of the aggregated cytosolic proteins, which are transferred to the mitochondrial matrix and are degraded by proteases^56^. For instance, inhibition of the signal recognition particle in the ER causes accumulation of secretory proteins in mitochondria^57^. Therefore, the mitochondrial ubiquitin-proteasome pathway appears to function in mitochondrial as well as cellular protein quality control.

As explained above, alphaproteobacteria are considered the ancestors of mitochondria, and they form vesicles to store virulence factors or peptides that arrest the cell cycle of the host cell to defend themselves^58^. Similarly, it has been reported that mitochondria generate vesicles called mitochondrial-derived vesicles (MDVs), which function as interorganelle communication shuttles^59^. MDVs are quite small (70–150 nm), and they can have double or single membranes without a cristae structure^59^. MDVs contain the OMM protein related to the mitochondrial fission-regulating protein called mitochondrial-anchored protein ligase (MAPL), which is associated with communication with peroxisomes^59^. However, there are MDVs lacking MAPL^60^, which indicates that MDVs are heterogeneous and play multiple roles. Recent reports have proposed that some MDVs are destined to lysosomes and participate in mitochondrial quality control. For example, under oxidative stress conditions, when mitochondrial damage occurs, the formation of MDVs is greatly increased, while their degradation is mediated by the lysosomal pathway^61^. The molecular mechanism by which oxidative stress triggers MDV formation is not yet well understood. PTEN-induced kinase 1 (PINK1) appears to play a significant role in MDV formation since its deletion prevents MDV formation^62^. Because PINK1 is also important for mitophagy, it remains unclear whether PINK1 contributes to MDV formation directly or indirectly via the modulation of mitophagy.

When massive and catastrophic mitochondrial damage occurs, the macroautophagic pathway selectively removes damaged mitochondria, a process referred to as mitophagy. Mitophagy requires multiple pathways and proceeds via three major steps: (1) isolating the damaged part of mitochondria from the mitochondrial network by enhancing mitochondrial fission, (2) tagging the impaired mitochondria with an “eat-me” signal, and (3) specifically removing the damaged mitochondria, which are engulfed by autophagosomes. PINK1 and Parkin are major proteins involved in the ubiquitin-dependent mitophagy pathway. In healthy mitochondria, PINK1 is localized in the mitochondrial matrix, where it is degraded in a proteasome-dependent manner. However, upon mitochondrial depolarization, PINK1 accumulates at the OMM, and its transport to the mitochondrial matrix is inhibited. PINK1 phosphorylates substrates such as the E3 ligase parkin, activating parkin-dependent substrate ubiquitination^63–66^. Subsequently, OMM proteins are ubiquitinated in a parkin-dependent manner and recruit autophagy receptors and/or proteasomal degradation machinery. Similarly, uncharacterized protein 4-nitrophenylphosphatase domain and nonneuronal SNAP25-like protein homolog 1 (NIPSNAP1) and NIPSNAP2, which are mitochondrial matrix proteins, accumulate in the OMM after mitochondrial depolarization and can serve as autophagy-related receptors^67^. In addition, a recent report showed that the IMM protein prohibitin 2 is exposed to the mitochondrial surface via inner membrane herniation^68^, suggesting that structural damage of the OMM can trigger mitophagy.

### The role of mitochondrial dynamics in the mitochondrial quality control system

Mitochondrial morphology is connected to mitochondrial function. Thus, the mitochondrial morphology changes in response to alterations to extra- or intra-cellular demands. For example, under energy-deficient conditions, increased autophagy activity increases the energy reserves and generates building blocks by recycling various cellular components, including mitochondria^69^. However, mitochondria, which are the primary site of energy generation, escape degradation in autophagosomes by increasing their length^70^. On the other hand, mitochondrial fragmentation occurs by increasing mitochondrial fission in response to stimuli promoting mitophagy, which accelerates selective mitochondrial degradation^71^. Therefore, disruption of fusion–fission dynamics, inducing extremely fragmented or hyperelongated mitochondrial morphologies, is associated with the mitochondrial quality control system and several diseases. For example, perturbation of mitochondrial fusion by genetic mutation causes autosomal dominant optic atrophy and Charcot–Marie–Tooth type 2A. In addition, excessive mitochondrial fission is observed in neurodegenerative diseases, such as Alzheimer’s and Parkinson’s disease^72^.

In most cases, morphological changes in mitochondria occur prior to the initiation of mitophagy^73–75^. This is a critical step because autophagosomes cannot engulf mitochondrial pieces greater than specific size^76–78^. For example, hyperglycemia induces mitochondrial fragmentation associated with mitochondrial membrane potential (MMP) loss through an increase in Drp1 activity, and the fragmented mitochondria are cleared by mitophagy to reduce energy production^71,79^. On the other hand, starvation causes mitochondrial elongation by inactivating Drp1 to prevent mitochondria from undergoing autophagic degradation^70^. Indeed, mitochondrial elongation through the downregulation of Drp1, accompanied by increased MMP, prevents mitophagy progression in cells^80^. Conversely, Drp1 overexpression decreases not only the MMP but also the mitochondrial mass through the induction of mitophagy^81^. Similarly, genetic knockout of Drp1 prevents mitophagy induction, resulting in lethal cardiac dysfunction in mice^80^. OMM-associated degradation, which mediates the proteasomal degradation of phosphorylated or ubiquitinated OMM proteins, also promotes mitophagy^82^. For example, Mfn1/2 are rapidly ubiquitinated via the PINK1/parkin pathway and are removed by the proteasome^83^. This process prevents damaged mitochondria from being incorporated into the healthy mitochondrial network. Therefore, coordinated changes in mitochondrial morphology and MMP are critical for mitophagy.

Several reports have questioned whether Drp1-dependent mitochondrial fission is required for mitophagy. For example, isolated autophagic membranes appear near mitochondrial regions and gradually extend along the mitochondria. At these sites, mitochondria start budding, and the isolated autophagic membrane enwraps these budded mitochondrial regions, which are eventually divided in a Drp1-independent manner^84^. Similarly, Youle’s group reported that mitophagy occurs normally, even in Drp1-deficient cells^85^. Although Drp1 seems to be dispensable for mitophagy, most reports support the notion that Drp1 plays a positive role in the regulation of mitophagy. For example, in a yeast model, the Drp1-like protein Dnm1 forms a complex with autophagy proteins Atg11–Atg32 to enable mitochondrial fission and mitophagy^84^. In mammalian cells, FUN14 domain-containing protein 1 (FUNDC1), which mediates parkin-independent mitophagy, interacts directly with both Drp1 and OPA1 at the OMM. Mitochondrial damage causes the disassembly of the FUNDC1–OPA1 complex and the recruitment of Drp1 to promote mitochondrial fission, coordinating the coupling of mitochondrial fission and mitophagy^86^.

Recently, we discovered that Drp1 directly interacts with the mitochondrial zinc transporter ZIP1^87^. Mitochondrial ZIP1 is also coupled to the MCU and Drp1–ZIP1 interactions that promote the influx of zinc ions into the mitochondrial matrix and cause a transient reduction in the MMP. Drp1–ZIP1 interactions only occur at mitochondrial fission sites, and transient MMP reduction occurs after Drp1 recruitment. After fission, most mitochondria readily recover MMP, except for a small subset, which is eliminated by mitophagy. Thus, it appears that mitochondria that are unable to recover MMP are eventually eliminated by mitophagy (Fig. 2). This process explains how mitochondrial fission instigated via Drp1 actively contributes to the elimination of damaged mitochondria^87,88^. Although we cannot exclude the possibility that Drp1 randomly cuts mitochondria for routine maintenance of mitochondrial quality, Drp1 can be recruited to mitochondrial sites with focally damaged spots that are selectively removed. Especially under normal conditions, when local MMP reduction is only marginal, Drp1-dependent MMP loss may serve as a means of enhancing the contrast between normal and impaired mitochondrial segments, ensuring that the dysfunctional mitochondrial fragments are correctly recognized by the mitophagy machinery.

Prolonged inhibition of Drp1–ZIP1 interactions causes the accumulation of dysfunctional mitochondria with a reduced ability to produce ATP and an increase in the production of reactive oxygen species, suggesting the importance of this process for the long-term integrity of mitochondria. However, Drp1-dependent mitochondrial fission appears to play a significant role in the maintenance of mitochondrial quality. For instance, while knocking out Drp1 in cells cannot completely prevent mitophagy^85^, it causes the accumulation of misfolded mitochondrial proteins. Thus, Drp1 may affect mitochondrial quality in two separate ways: (1) Drp1-dependent mitochondrial fission prevents the diffusion of damaged molecules into the interconnected mitochondrial network, and (2) the interaction between Drp1 and ZIP1 promotes the segregation of damaged mitochondrial segments for selective mitophagy.

### Perspective and conclusion

Considering that mitochondrial fusion/fission machinery is derived from cytosolic endocytosed molecules, it is not surprising that molecules associated with mitochondrial dynamics also contribute to other cellular functions. While the role of fission/fusion molecules in the regulation of mitochondrial dynamics is well characterized, their contributions to the structural and functional dynamics of other organelles have only recently been recognized. For instance, in addition to mitochondria, Drp1 receptors are widely found in other membranous organelles, including peroxisomes and trafficking vesicles^89,90^. There is less evidence for their localization to the cytoplasmic membrane, but the localization of Drp1 to lamellipodia has been reported^91^. In addition, it has been reported that Drp1 variants localize to lysosomes, late endosomes, and the plasma membrane^92^. It is likely that the widespread localization of Drp1 receptors causes the wide distribution of Drp1, although further study is needed to identify the specific proteins that recruit Drp1 to different cell locations. This supposition is also supported by the fact that proteins associated with Drp1 contribute to actin bundling and lamellipodia formation^91^. Similarly, molecules responsible for mitophagy play additional roles in cell biology^89,91–93^. Thus, it is important to consider that any effects observed after the perturbation of these molecules may be caused by alterations other than those involved in mitochondrial dynamics/mitophagy.

Different subcellular compartments have make different demands on mitochondria; therefore, it is very important to consider that a cell has many mitochondria of various structures and functions^94–96^. Thus, it is impossible to simplify and address mitochondrial responses at the whole cell level, and close observation of the behavior of individual mitochondria within the cell is required. Recent advances in live cell imaging by superresolution microscopy and organelle interactomics approaches support the hypothesis that organelles within a cell interact dynamically and exchange information^97^. In fact, mitophagy is a process that exemplifies these interactions, as autophagosomes are produced in the ER, and the contribution of lysosomes to mitophagy is well understood. Thus, further studies of entire organelles within a cell should be conducted with high-resolution imaging, and systemic approaches will shed new light on the precise mechanisms by which organelle-level interactions contribute to mitochondrial structural and functional alterations.

One unexpected finding that provides a new perspective on mitochondrial biology is based on the observation that these interesting organelles can be secreted from the cell and can be transferred to neighboring cells^98–100^. As we explained in the beginning of this review, mitochondria originated evolutionarily from independently living bacteria. In this respect, mitochondria may maintain some features of their ancestral ability to survive extracellularly and infect host cells. Thus, it is tempting to speculate that mitochondrial biology and quality control systems are at the basis of the evolutionary origins of mitochondria, which can provide valuable insights about mitochondrial control.
